# Maximizing the Benefit of Respite for Dementia Caregivers: A Study Protocol Describing the Development & Evaluation of the Time for Living & Caring (TLC) Intervention

**DOI:** 10.21926/obm.icm.2304040

**Published:** 2023-10-08

**Authors:** Rebecca L. Utz, Michael Caserta, Eli Iacob, Catharine Sparks, Louisa Stark, Alexandra Terrill, Amber Thompson, Bob Wong

**Affiliations:** 1. University of Utah, College of Social and Behavioral Sciences, 260 South Central Campus Drive, Salt Lake City, UT, USA; 2. University of Utah, College of Nursing, 10 S. 2000 E., Salt Lake City, UT, USA; 3. University of Utah, School of Medicine, 27 S. Mario Capecchi Dr, Salt Lake City, UT, USA; 4. University of Utah, College of Health, 15 N 2030 East, Salt Lake City, UT, USA

**Keywords:** Respite, caregiver, online intervention, community-engaged research, protocol

## Abstract

Dementia caregivers are susceptible to adverse physical and mental health outcomes, given the often prolonged and challenging care and support they provide to family members with Alzheimer’s Disease and Related Dementias (ADRD). This report describes a community-engaged implementation of a novel behavioral intervention - an “app” (interactive website) called TLC (Time for Living and Caring) that coaches caregivers on how to maximize the benefits associated with respite time. The rationale and features of the TLC intervention and the full research protocol used to develop and then evaluate its feasibility, acceptability, and initial efficacy are described here.

## 1. Introduction

More than 11 million Americans provide unpaid care to family members or friends, at an estimated value of $340 billion annually [1]. Family or informal caregivers provide instrumental support and provide technical medical care, often without adequate support or training and usually without any compensation [2]. Caregivers often report considerable stress and burden, including financial hardships, making them at-risk for poor socio-emotional and physical health outcomes [3, 4]. Those caring for someone with Alzheimer’s Disease or Related Dementia (ADRD) report exceptionally high levels of daily stress [5], given the challenging symptoms and extended nature of the AD/ADRD illness [6].

Establishing ways to support family caregivers, especially ADRD caregivers, is critical to addressing their health and well-being, as well as recognizing the public health relevance of supporting family caregivers. The first major research-based interventions for caregivers included REACH I (1996–2000) [7], consisting of nine interventions to reduce AD/ADRD caregiver stress, followed by the 5-site REACH II studies (2001–2006) [8, 9]. These studies, as well as many others since then, have developed and evaluated various interventions related to self-care, safety, social support, emotional wellbeing, management of behavioral problems, skill training, telephone-based support, behavior modification, family therapy, computerized telephone communication, coping classes, and support groups [10–13]. Most caregiver support interventions receive high participant satisfaction, yet produce modest benefits for caregivers [14, 15].

Very few of the existing caregiver interventions have focused on respite [16–18]. Respite is commonly identified as the most needed and desired services for caregivers [19, 20], and has been said to be the most promising way to maintain and enhance caregiver wellbeing over time [21]. Respite is defined as time away from their caregiving duties [22]. Respite can be provided as a formal service by an adult day center or in-home respite providers, or in institutional or overnight settings; respite may also be provided informally to a caregiver through the tag-teaming and shared arrangements that families, friends, and neighbors set-up to ensure that the care-recipient’s needs are fully taken care of and that the primary caregiver gets an occasional break. When scheduled regularly and in sufficient doses [23, 24], respite gives caregivers a temporary break to tend to their own health, maintain social and family relationships, and pursue aspects of their daily lives that they may have neglected as a result of the caregiving demands [25]. Policy initiatives, including the Recognize, Assist, Include, Support, and Engage (RAISE) Family Caregivers Act [26], the 2022 National Caregiver Strategy (https://acl.gov/CaregiverStrategy) and the 2023 White House Executive Order (#14095), all suggest that respite is an essential component of caregiver support.

Research finds inconsistent or mixed results on the overall benefit of respite to caregivers [22, 23, 27, 28]. Almost half of caregivers reported dissatisfaction with how they spent their respite time; many reported “wasting” time doing lower priority activities, instead of using their respite as a reprieve from their role as vigilant caregivers to pursue activities that are personally meaningful or rewarding [19]. Respite research has typically focused on a comparison of respite users to non-users, without consideration of what caregivers do during their respite time. Using time-use data, we found that those who used respite to do what they had most desired, needed, or had planned to do had the highest evaluative assessments (satisfaction) with their respite time-use and also reported the most positive wellbeing over time (i.e., lower levels of burden and depressive symptoms) [19, 20, 29]. These findings emerged, regardless of what kind of activity they chose to do during respite. Caregivers benefited whether they vacuumed the house or went golfing with a friend - IF the activity was what they had planned or desired to do. This finding is consistent with time-use research, in general, which argues that congruence between desired and actual time-use is a significant predictor of overall life satisfaction [30]. As described in Box 1, we created an intervention to address and support the time-use needs of family caregivers. This report describes the intervention.

## 2. Description of the TLC Intervention

The name of the TLC intervention - Time for Living and Caring-implies that caregivers need and deserve to take time for their personal lives in addition to their caregiving responsibilities. Initial pilot tests of the TLC intervention suggested high participant satisfaction and provided preliminary evidence of efficacy as a potential intervention model for caregivers [29]. However, as originally conceptualized and piloted, TLC was delivered by trained facilitators who made as many as 15–20 visits or phone calls with individual caregivers to ensure treatment fidelity of the intervention. This type of labor-intensive delivery approach has obvious challenges for scalability and implementation.

Consistent with recommendations from a National Institutes of Health research summit [31], widely available mobile and internet technologies hold promise in delivering effective and cost-efficient interventions, even among the older users [15, 18, 32, 33]. While online-delivered interventions or “apps” may not be suitable or preferable for all caregivers, especially for those without access to high-speed internet or computer technology and those who are not interested in engaging with these types of technologies [34], such limitations are likely outweighed by the strengths, particularly their ability to deliver support to hard-to-reach populations, such as caregivers who may not be available to engage in educational or supportive services during normal business hours and those who live in rural and remote areas where traditional in-person educational and supportive services may not be available.

The TLC intervention consists of an *Initial Assessment*, followed by a series of *Goal-Setting*, and *Goal-Review* activities designed to help caregivers better plan their respite time-use. The was designed to reflect the theoretical principles of “*Selective Optimization with Compensation,*” [35, 36], a well-researched theory from social psychology and gerontology that describes how people often adapt to age-related changing circumstances and capacities by employing the three strategies of selection, optimization, and compensation. The online version of the TLC intervention uses an interactive calendar and a virtual or automated coach (i.e., pop-up instructions and guided prompts) to help caregivers plan and schedule their respite time.

The TLC intervention begins with an *Initial Assessment* of one’s individual needs, circumstances, priorities, and resources - as they relate to respite. The assessment first asks whether the caregiver feels they are getting enough respite time and how much time-off they would like to have each week. These initial questions also help the caregiver identify specific activities once enjoyed but potentially sacrificed due to caregiving responsibilities, and to brainstorm “wish list” activities they would like to do but have not had time to accomplish because of their caregiving responsibilities. These initial questions and activity list, self-generated by each person, raise self-awareness about respite time-use and therefore prime the caregiver to be able to engage in the goal setting activity.

Next, after the Initial Assessment is completed, a Goal-Setting module is activated. Again, in the form of pop-up instructions and guided prompts, the virtual coach reminds caregivers to schedule their upcoming respite time on the interactive calendar. Then, for each scheduled block of respite time marked on the calendar, the virtual coach guides the caregiver through a series of short questions that help them identify, define, and potentially modify their respite time-use goals. A modified “SMART goals” framework (defined as goals that are specific, measurable, attainable, relevant, timely) [37, 38] provides a framework to guide caregivers through the process of goal setting or goal selection, and facilitates the three theoretical adaptive strategies: first, caregivers identify and prioritize which of the specific possible activities they listed in the Initial Assessment they would like to do during a specific respite time-block (*selection of goals*); Automated pop-up messaging then reminds them to think about any potential limitations in the time available or resources available to accomplish that activity (need for *compensation*), and to consider ways they might remove obstacles to make goal attainment more likely (facilitates *optimization).* Engaging in the conscious process of *selection- compensation-optimization* requires prioritizing [36] and oftentimes points out the need for advance planning to ensure that the goal or activity can take place during a future scheduled time-block when respite is available, especially if the desired time-use activities involve, for example, the participation of others [39], making advanced reservations and appointments, or other compensation strategies that might be required to accommodate one’s personal circumstances. Two scenarios, below in Box 2, offer an illustration of how the theoretically-informed goal-setting process unfolds; these are actual examples of how respite time-use planning unfolded with two older adults who were initial users/participants of TLC:

A meta-analysis of 94 studies revealed that having a clearly stated goal and a realistic plan of action was positively related to successful goal attainment [40]. Other studies find evidence that the process of goal-setting can facilitate behavior change even when the person may be reluctant, [41] because the process of specifying goals and developing realistic and individualized implementation plans increases self-awareness and has positive impacts on subsequent behavior [41–44].

The third component of the TLC intervention is Goal-Review. Each week, the caregiver is prompted by the app to reflect on the extent to which they met their stated respite time-use goals in the previous week. The American Time Use Study (ATUS) describes two components of time-use - experienced and evaluative. Experienced time-use measures “momentary positive and rewarding or negative and distressing states,” while evaluative assessments of time-use refer to how activities shape caregivers’ “judgments of their overall life satisfaction or dissatisfaction” [45]. Suppose that a caregiver utilizes respite time to go to the grocery. The caregiver might experience some relief because they were able to get out of the house and their focus was taken away at least momentarily from caregiving tasks. However, the caregiver could also feel dissatisfied because grocery shopping may feel like an obligation and not much of a break from caregiving. Previous research with caregivers found that a broad range of activities, both obligatory (e.g., employment or cleaning the house) and discretionary (e.g., leisure or social events), were associated with positive assessments of time-use [19, 20]. Interpreting these data from the lens of the experienced versus evaluative time-use revealed a positive correlation between time-use and well-being, driven by whether the chosen activity (regardless of whether it was obligatory or discretionary) was what caregivers had *desired or planned* to do during respite. Thus, the primary purpose of the goal-review process is to ascertain whether the caregiver did the activity they had planned to do during their scheduled respite time. The caregivers’ goal-review responses - both experienced and evaluative time-use assessments- are recorded on a visual *dashboard*.

The virtual coach provides prompts to help the caregiver repeat the goal-setting and goal-review process each week, by asking whether the caregiver would like to identify new goals, repeat existing ones, or revise former goals using the same assessment and goal setting strategies that were used previously. The act of calendaring respite time raises self-awareness of how much respite time one had. Then, explicitly planning what kinds of activities they want to do during their respite time, followed by reviewing whether they were able to do what they had planned to do, raises their self-awareness about how useful and meaningful their respite time-use was. In other words, the goal-setting and goal-review process allows them to reflect on both their experienced and evaluative time-use. Over time, caregivers are expected to become more self-aware and independent in their respite planning [46, 47]. And, respite planning may maximize the benefit of respite, thereby allowing caregivers to maintain their well-being over time.

Note: the virtual coaching prompts asked caregivers to plan and reflect on their weekly time-use goals, rather than doing a 24-hour diary approach that is commonly used in time-use studies. This reduces the burden associated with collecting traditional time-use data -a critically important methodological modification for a highly burdened population like caregivers.

The online version of the TLC intervention also includes general information and educational resources to help caregivers further maximize the benefits of respite. Educational modules included:

1. *What is Respite? a*n information page that provided an introductory video describing the benefits of respite and the importance of scheduling regular respite,
2. *How do I Get Respite*? a page that provides numerous links to service providers and reimbursement information about respite, resources about how to choose the right respite provider, and guides on how to work with family members, neighbors, and friends to provide informal respite, and
3. *Take a Break - What is Holding you Back?”* a library of resources intended to help caregivers prepare for respite, including resources to guide caregivers on what to tell care recipient prior to a respite provider coming, a fillable worksheet that could be left with respite providers to describe the kind of care required while the caregiver has respite, and a recorded meditation exercise intended to help caregivers “reset” before respite and let go of any guilt or anxiety about taking respite.

While not originally planned, the TLC app ended up including a fourth information module that provided caregivers with lists of respite ideas and activities that could be done in the home, oftentimes with the care-recipient present. These ideas were necessary and reflected the unique historical moment in which the TLC app was launched (late spring/summer of 2020), a time when many of the traditional respite providers as well as the informal arrangements made with friends and family to provide shared caregiving became unavailable because of the COVID-19 pandemic [48–53].

## 3. Materials and Methods

### 3.1 Study Overview

**Figure 1:**
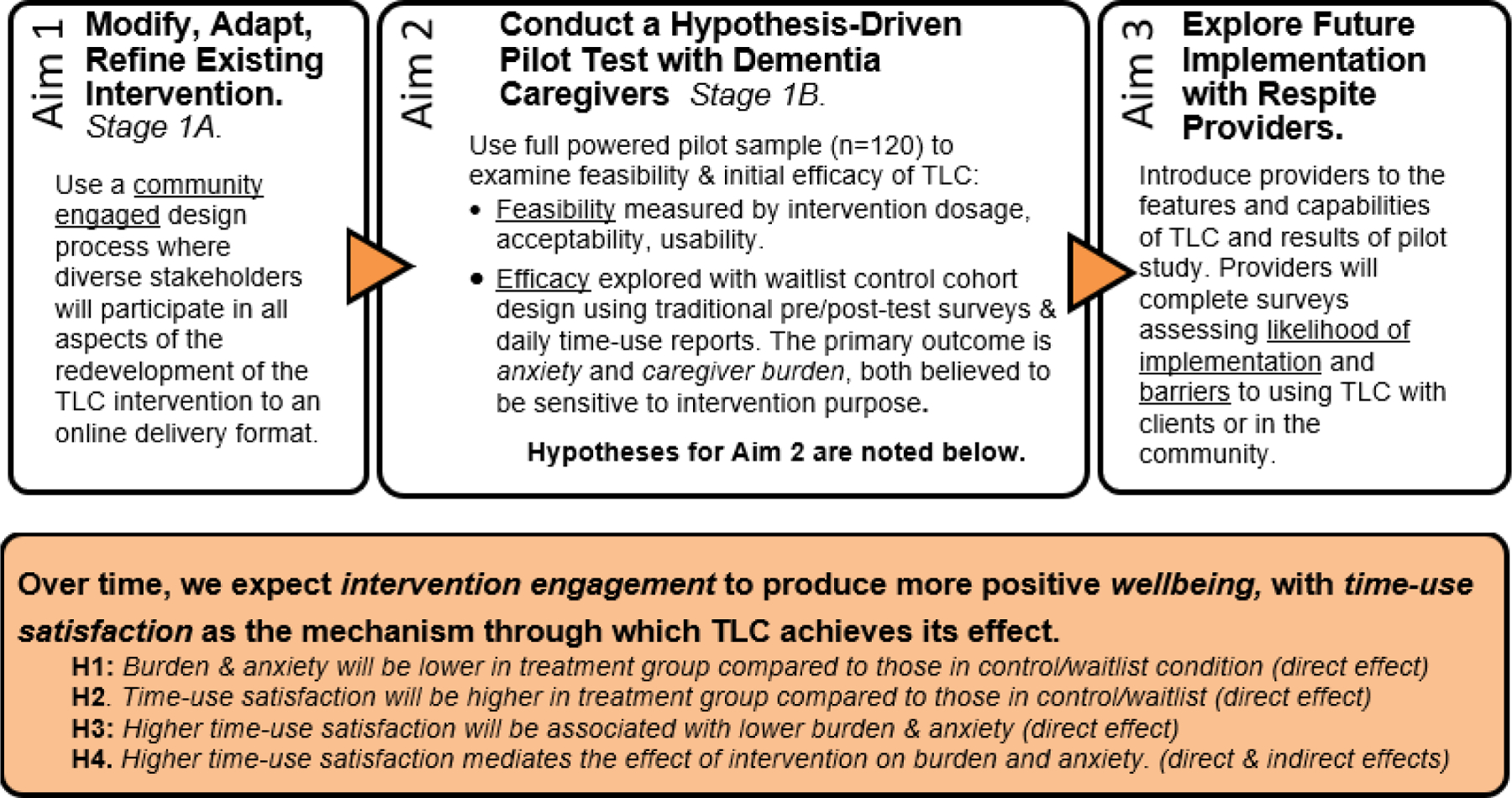
TLC Study Aims & Hypotheses.

As shown in Figure 1, the TLC research study was created to guide the intervention (re)development and the evaluation of its implementation with a community-based sample of ADRD caregivers. The TLC Study consisted of a set of comprehensive research activities coinciding with the pilot-testing stage (Stage 1) of the NIA stage-model for behavioral interventions [54, 55]. Aim 1 used a community-engaged design process to redevelop and refine the original TLC intervention into a self-administered, web-based delivery platform (Stage 1A) [56]. Aim 2 relied on the recruitment of a fully-powered pilot sample of dementia caregivers and a randomized clinical trials study design to examine the 1) feasibility, usability, and acceptability of the TLC intervention, 2) the efficacy of the TLC intervention on caregiver well-being outcomes overtime, and 3) hypothesis testing to help identify and isolate “time-use satisfaction” as a mechanism through which the intervention achieved its effect on caregiver well-being (Stage 1B). Aim 3 consisted of a nationwide survey of respite providers, who offered an additional layer of descriptive data regarding the feasibility and usability of the intervention, with a particular focus on its potential implementation and translation through existing respite provider networks. The methodology and outcomes of Aim 1 are described elsewhere [56]; the work associated with Aim 3 is ongoing; and this report focuses on the description of the protocols and procedures associated with Aim 2. All study procedures and protocols were approved by the Institutional Review Board at the University of Utah.

### 3.2 Study Design

**Figure 2:**
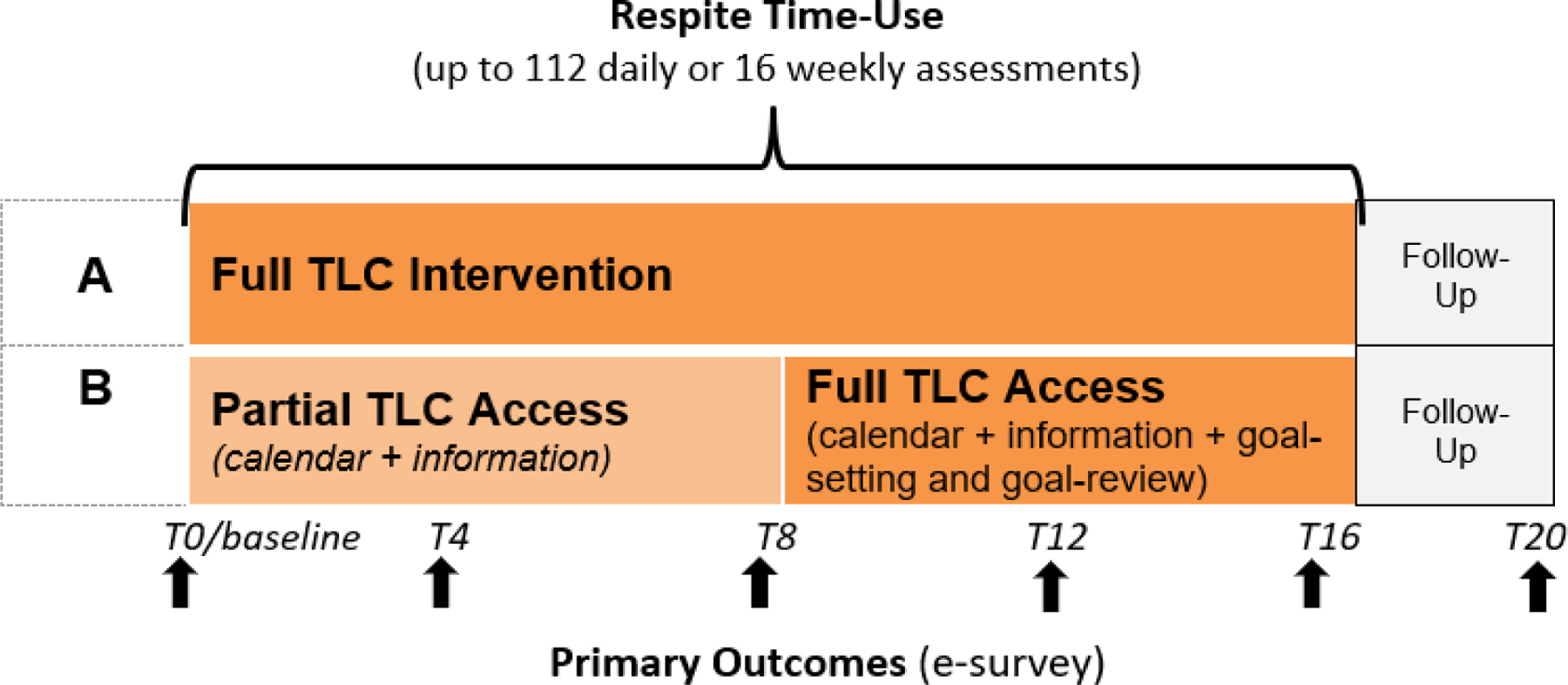
TLC Study Design. Note: Pilot data have already been collected, at the time of publication. Refer to Section 5.

The TLC research study utilized a randomized waitlist-control longitudinal cohort design, such that all participants received the full set of tools associated with the TLC intervention. As shown in Figure 2, participants were randomized to one of two intervention sequences:

Group A received access to the full TLC intervention, including the information/education modules, the interactive calendar to schedule respite, and the weekly virtual coaching prompts (goal-setting and goal-review) for the entire 16-week intervention period. They were encouraged to engage with the app at least once per week.

Group B had 8 weeks of partial access, followed by full access for second 8 weeks. During the modified “waitlist” period (i.e. weeks 1–8 of the intervention), caregivers only had access to the information pages and the calendar where they could schedule and document their respite time, and weren’t given full access to the weekly coaching modules (goal-setting and goal-review) until weeks 9–16.

The purpose of this two-group design allowed us to evaluate whether caregivers benefitted from gaining full access to the intervention materials and functions of the virtual coach from the start, or whether they preferred or benefitted from a more gradual introduction where they first practiced scheduling respite and getting a more consistent respite schedule before they were guided through the goal-setting and goal-review exercises to plan what they wanted to do during that respite time. This modified wait-list control type of design allowed us to isolate whether the goal-setting and goal-review components of the intervention were the key mechanisms that allowed caregivers to improve their respite time-use and time-use satisfaction, as hypothesized.

Information recorded within the TLC app (i.e., on the calendar and/or obtained through the goal-setting and goal-review modules) provided repeated measures of respite time-use for a total of 16 weekly or up to 112 daily reports. Primary and secondary outcomes data were collected via electronically administered surveys at baseline (T0) and every 4 weeks thereafter (T4, T8, T12, T16, T20), for a total of 6 longitudinal assessments. This randomized, prospective, and longitudinal design allowed each participant to serve as his/her own control and offered opportunities to assess both within- and between-group differences over time [22].

### 3.3 Sample and Eligibility Criteria

Eligibility for the TLC research study was limited to ADRD caregivers who had access to respite (formal or informal) at least once a week for a minimum of 4 hours. Research has found that respite should be used on a regular basis and in sufficient doses to be effective [23, 24]; thus, it is a reasonable expectation that participants should have minimum and regular respite usage patterns, even if the intervention provided encouragement and resources to help caregivers increase their overall access to respite over time.

Additional eligibility criteria included: 1) being the primary caregiver (defined as the family member who performs the majority of the caregiving tasks), 2) co-residing with the care recipient, 3) being age 18 or over, 4) ability to read and follow instructions written in English (because the app was developed only in English language for this pilot study), and 5) providing care to someone with cognitive impairment and/or a diagnosis of AD or ADRD, as self-reported by the caregiver. If necessary, research staff asked screening questions related to the common symptoms of AD, as described by an NIA factsheet (Pub No 15–6423), including the presence of at least two reported symptoms: memory problems, wandering, difficulty with money, and noticeable personality changes [57]. Since females comprise about 67% of family caregivers overall [1], we expected a female-dominated sample; however, purposive, convenience recruitment and sampling strategies targeted men as well as racially and ethnically diverse caregivers.

### 3.4 Recruitment

Participants for the TLC study were recruited using our established relationships with local community partners, including Community Faces of Utah, an established and unique university-research partnership that provided assistance and guidance in recruiting diverse family caregivers from Pacific Islander, African American, Hispanic, Native American, and refugee communities; the Utah Telehealth Network, who assisted us in reaching caregivers living in the rural areas of the state; and the Utah Coalition for Caregiver Support, who provided us with access to caregivers and respite users residing primarily in the urban and suburban areas of Salt Lake, Weber, Davis, and Utah counties. We also recruited through local geriatric and neurology clinics that were willing to distribute study materials to potentially eligible patients. Local organizations that sponsored caregiver support programs and services also provided referrals of potentially eligible participants. Finally, we used local newspapers and other community-based forums, including Facebook, to advertise the study. This multi-pronged, community-based recruitment method primarily produced referrals, which were individually screened (via phone and video conference) by TLC study staff.

Altogether, we wanted to recruit and consent a total of about 150 participants, with an assumption that there may be as much as 25% attrition rate, resulting in an analytic sample of at least 120 caregivers. Power calculations suggest that, even after controlling for projected attrition, this is a fully-powered pilot sample [58]. The TLC sample has 163 participants, with roughly half randomized to Group A and half to Group B.

### 3.5 Consent and Enrollment

Once a potentially eligible caregiver was identified or referred to the project, the TLC Project Manager and/or Research Assistant followed recommended best-practices for enrollment and consent [59]; they conducted follow-up call with each referred caregiver to formally screen eligibility and obtain formal verbal consent. An orientation visit (usually conducted as a teleconference) provided an introduction to the study, as well as brief screening of their personal technology and internet access. We encouraged participants to use their own technology, if possible, to access the TLC intervention, since that would provide us with more realistic information about the usability and feasibility of the intervention as accessed on a number of different types of devices, browsers, and networks. However, when needed, the project provided a laptop and/or high-speed internet service to those persons who did not have reliable access to these technologies, but wanted to participate in the study.

According to recent data, Utah as well as other parts of the United States have high rates of internet and personal computer access, even in the rural and frontier regions of the state: 95% of households have a computer and 86% have high-speed internet access [60]. Although we assume that the enrolled participants are likely a more tech-savvy set of caregivers than the average caregiver population (given the nature of the study), participants with any level of computer and technology experience were encouraged to participate. TLC project staff were available throughout the study period to provide additional encouragement and personalized technical support, as needed or desired. Thus, as part of our feasibility assessment, we will capture participants’ comfort with the online delivery and will record whether technology-restrictions or aversions were a factor in recruitment, retention, attrition, or usability and feasibility of the intervention itself.

### 3.6 Retention

Retention efforts are as critical in achieving a high-quality sample and data. The longitudinal clinical trials study design requires consistent engagement and participation over a 16–20 week time period, which may be challenging for ADRD caregivers who are typically facing time-consuming caregiving demands. To encourage continued participation, we provided each participant with a prorated compensation package, totaling around $250 if they participated in all aspects of the study. Participants could choose if this was paid in the form of gift cards or in the form of a new Dell Chromebook (laptop).

Other retention efforts included providing participants with caring and personalized support from research staff. A key factor in recruitment and retention is a professional staff that demonstrates empathy, establishes rapport, and maintains regular contact with the participants [59]. Personalized contacts were most often initiated by participants, in response to comments or question about the technology or functionality of the TLC app, while some contacts were initiated by our staff if we identified a participant that was having a difficult time with the technology or with life in general (most often, identified through staff-review of submitted questionnaires). A clinical psychologist provided training and ongoing consultation with the study personnel responsible for recruiting and retaining study participants. The clinical psychologist also prepared resource sheets for those who needed additional mental health resources or other supports in the community beyond the scope of what we could provide in the study.

**Figure 3:**
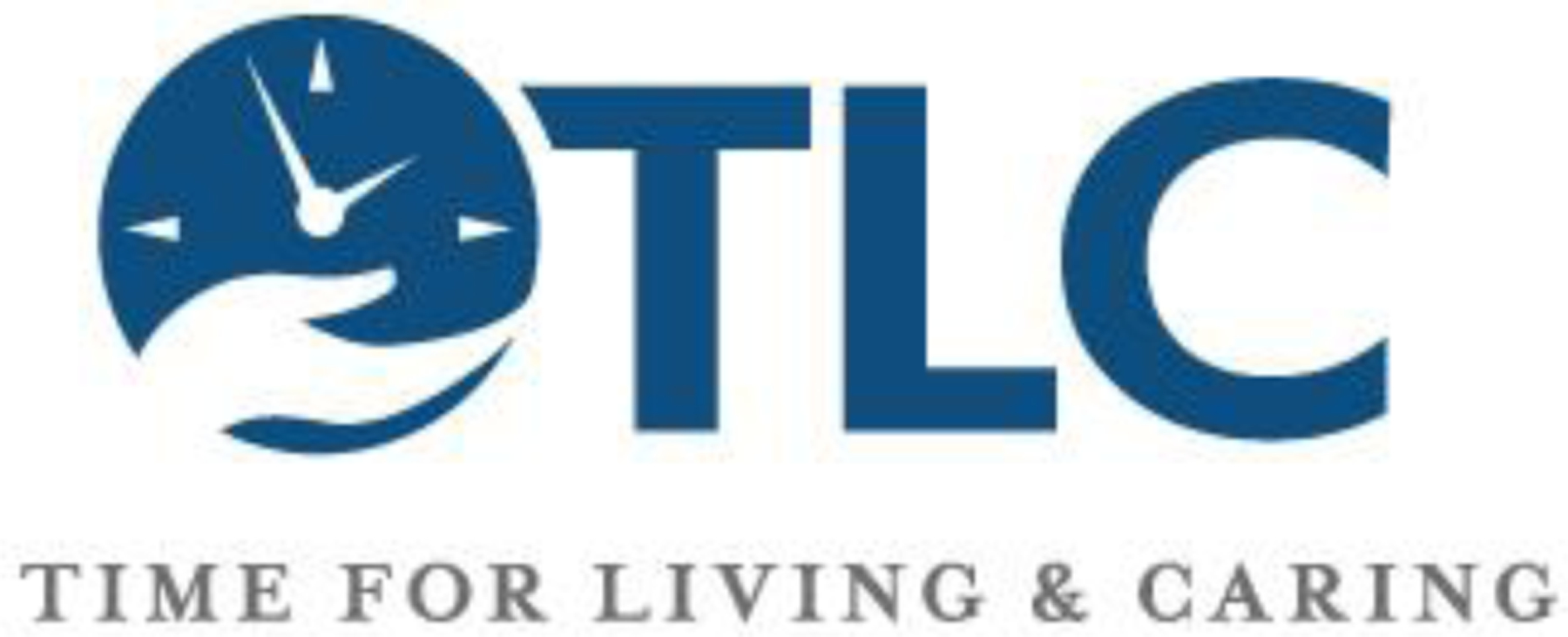
TLC logo.

Finally, the TLC study sent handwritten notes, signed by the members of our study team, once at the start of the study (along with a magnet), thanking them for agreeing to be part of the study, and once when each participant was about half-way through the intervention period, thanking them for their continued participation. The second note was accompanied by a small gift package including a TLC-branded journal, pen, and a deck of playing cards, along with an informational sheet that provided tips on how to journal or play solitaire as a mini respite break during their day. All materials related to TLC had a similar branding (i.e., color, logo, imagery), which required an investment in graphic design and production. The TLC logo is shown below in Figure 3.

### 3.7 Data Collection and Measures

**Figure 4:**
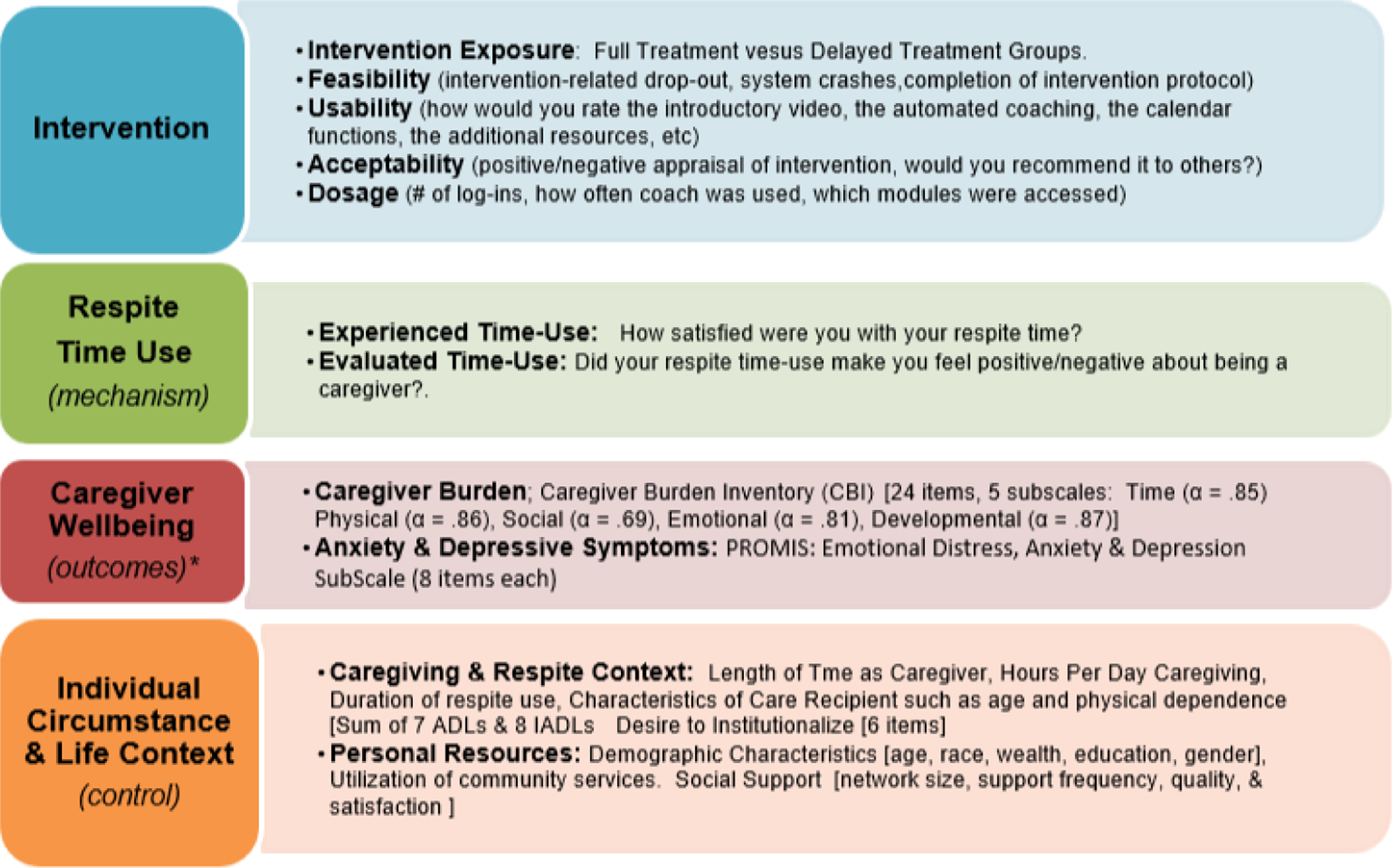
Measurement of Key Constructs. Notes: *caregiver well-being outcomes were also measured using positive affect, positive aspects of caregiving scales. Sources of Measures: [18, 19, 61–71] and http://healthmeasures.net.

All data needed for analyses were collected from the caregiver respondents using e-survey methodology. Figure 4 describes the key constructs and variables measured in the TLC research study, along with notes about when and how often each measure was collected. Chosen measures have sound psychometric properties, proven validity, and theoretical relevance to the intervention’s purpose and its hypothesized mechanism of action.

### 3.8 Planned Analyses

The TLC study employed two primary analytic strategies - the first set of analyses use the full pilot sample to descriptively report variations in how study participants accessed and used the intervention materials and whether our research protocols were feasible, while the second set of analyses used the same sample, yet focused on the inferential relationships and hypothesis testing.

#### 3.8.1 Evaluation of Feasibility, Acceptablity, and Usability

Planned analyses include reporting univariate statistics such as frequency, central tendency (mean, median, mode), ranges, and standard deviation for the following measures: *feasibility, usability, acceptability* and *dosage*. Table 1 illustrates examples of quantifiable indicators developed for each outcome. Analyses will be used to assess our research procedures and protocols to ensure we are adopting best-practices for clinical trials designs and for the testing of technology-delivered interventions. We will use the feasibility, usability, and acceptability measures to learn how participants used the TLC app, giving us insight into how the app might be refined in the future to better accommodate the needs, preferences, and uses among caregivers themselves.

#### 3.8.2 Hypothesis Testing; Evaluation of Intervention Efficacy

**Figure 5:**
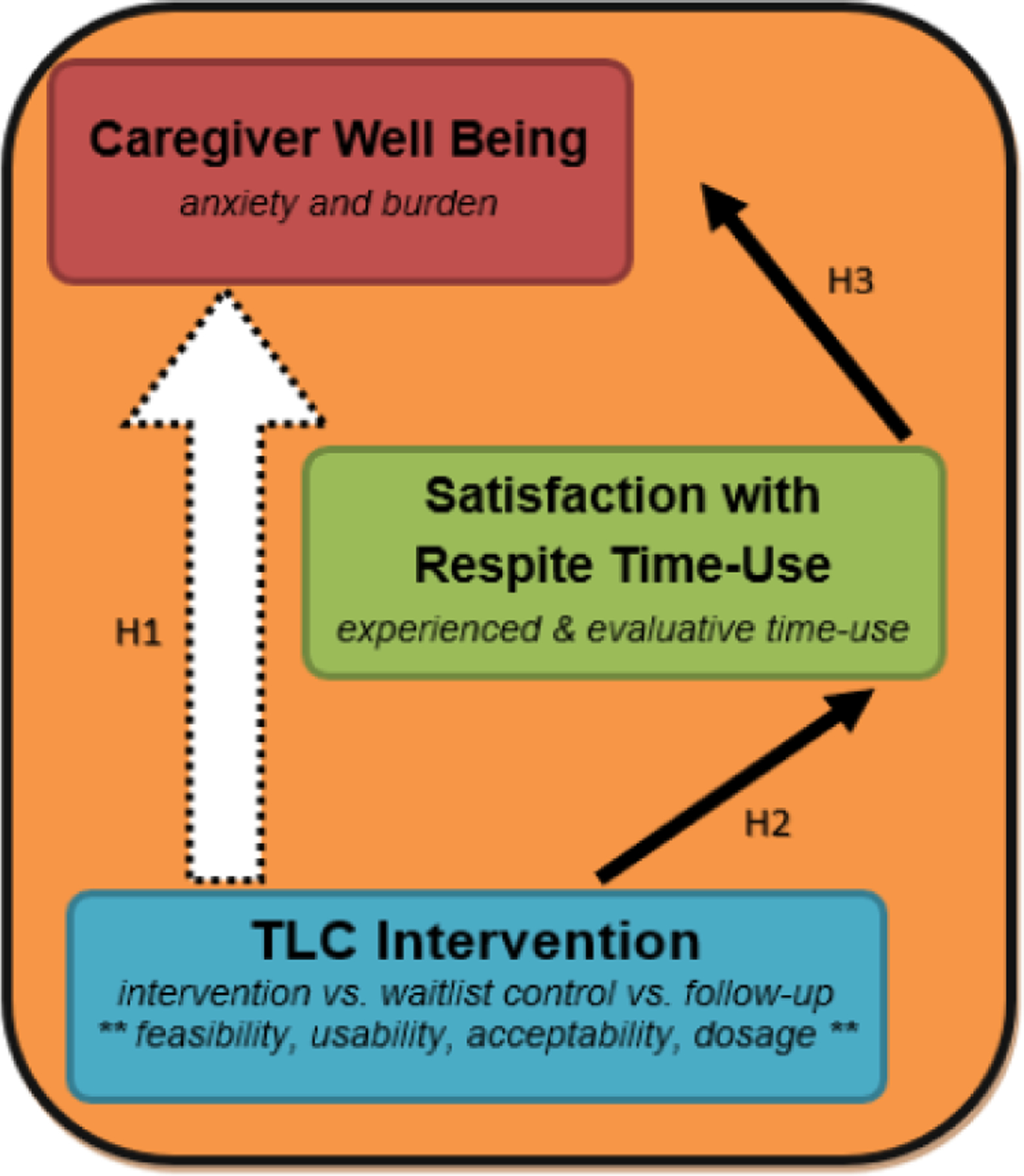
Conceptual Model Underlying Hypotheses and Analyses of Caregiver-Reported Outcomes.

The second set of analyses leverage the within-and between-person comparisons afforded by the waitlist control cohort design to assess *efficacy* of the TLC intervention. The focus of these analyses are on caregiver-reported outcomes. The primary analyses are statistical in nature and align with the conceptual model outlined in Figure 5. It is assumed that the TLC intervention might lead to improved wellbeing over time (**H1**), as shown by the large white arrow. An effective intervention would have the potential to improve caregiver wellbeing, typically measured with “distal” or global outcomes of depressive symptoms, life satisfaction, happiness, and physical/functional health [7, 72–76]. In practice, however, there are relatively few studies demonstrating that interventions are able to significantly or sustainably modify these types of global measures over time [61]. Thus, designing a study focuses on the global or distal measures of wellbeing would be shortsighted and at risk of reporting no treatment effects. To avoid an overreliance on measures that may be hard to modify, we focused on two wellbeing outcomes that are more proximal or specific to the caregiving and respite context [77], and thus are expected to be more responsive to the intervention-*anxiety* and *caregiver burden*.

Furthermore, our conceptual model has an indirect pathway through which, we hypothesize, that the intervention may achieve its effect on caregiver wellbeing. As described earlier, the TLC intervention prompts caregivers to engage in assessment, goal-setting, and goal-review strategies in order to improve their self-awareness and overall experience and evaluation of their respite time-use. The black arrows in the figure that link the intervention to an increase in time-use satisfaction (**H2**), and then increased time-use satisfaction with more favorable caregiver wellbeing (**H3**) describe the pathway or mechanism through which the intervention is thought to achieve its effect on wellbeing. “Satisfaction with Respite Time-Use” is the name of the construct we use to describe the concepts of *experienced* and *evaluative time-use*. Thus, our final analyses will compare the direct and indirect pathways represented in this model: To what extent does time-use satisfaction mediate (or partially explain) the effect of the TLC intervention on caregiver wellbeing. Together, these analyses allow systematic exploration of intervention *efficacy*, as well as potential identification of the *mechanism* through which the intervention achieves its effect [78]. Finally, it is important to note the large orange box encircling this conceptual model; this represents characteristics, resources, and life circumstances of each individual. Considering these *contextual influences* is critically important given the social determinants of health and may affect any of the pathways depicted in the model over time.

Guided by this conceptual model, analyses ask: 1) *What happens?* W*hat is the magnitude of the overall effect?* and 2) *How does it happen? What are the potential mediating pathways, and how strong are they*? Conventional clinical trials consider primarily the first question only, but we extend the standard approach with planned statistical analyses that can investigate the mechanism of change that produces outcome efficacy [78]. This is a key goal for early-stage intervention research, as it allows future intervention refinements to address the specific components of the intervention that are most impactful and lead to meaningful change in desired outcomes. The modified waitlist-control design, where Group B receives incremental access to the features and functions of the TLC intervention, is particularly suited to assess whether the goal-setting and goal-review activities are the key ingredient or mechanism driving the intervention effect.

Analyses focuses on testing four primary hypotheses related to how the intervention affected caregiver-related outcomes; refer back to Figure 1. Hypothesis testing depends on multivariate relationships, which are efficiently estimated using generalized linear mixed modeling [79]. This framework extends naturally to additional predictors and covariates, and permits more precise estimation of individual therapeutic gain by separating measurement error from true change. For randomization group (*g*), the full structural equation model is

(1) $$\begin{array}{c} {\text{y}}^{\left(\text{g}\right)}=\text{τ}+\text{Λ}{\text{η}}^{\left(\text{g}\right)}+{\text{ε}}^{\left(\text{g}\right)},{\text{ε}}^{\left(\text{g}\right)}\sim \text{MVN}\left(0,\text{Θ}\right);\;\;{\text{η}}^{\left(\text{g}\right)}={\text{α}}^{\left(\text{g}\right)}+{\text{Β}}^{\left(\text{g}\right)}{\text{η}}^{\left(\text{g}\right)}+{\text{ζ}}^{\left(\text{g}\right)},\;{\text{ζ}}^{\left(\text{g}\right)}\sim \text{MVN}\left(0,{\text{Ψ}}^{\left(\text{g}\right)}\right) \end{array}$$

where y is the vector of observed outcome variables at pre-treatment and follow-up, τ contains the (invariant) intercepts of the observed assessments, Λ is the (invariant) matrix incorporating design and multiple indicator measurement relationships, η is the vector of latent constructs, having unrestricted covariance matrix Ψ, Β are regression coefficients among latent variables corresponding to explanatory pathways, ε are measurement errors at each occasion, and α contains the latent variable means at each occasion.

Hypotheses H1 and H2 evaluate whether the impact of the full intervention (TLC-Group A) on Respite Time-Use & Wellbeing differs from that provided by the wait-list control (WLC-Group B). Similarly, hypothesis H3 evaluates whether respite time-use affects wellbeing. These relationships adopt an intent-to-treat comparison of outcomes and is therefore valid under any set of intervening paths or mechanisms. The null hypotheses tests outcome Y at particular times and change across time, conditioned on baseline values.


(2) $${\text{H}}_{0}:\;\;\text{E}\left({\text{Y}}_{\text{TLC}},\;\text{time}/{\text{Y}}_{\text{baseline}}\right)=\;\text{E}\left({\text{Y}}_{\text{WLC}},\text{time}/{\text{Y}}_{\text{baseline}}\right)$$

This is the conventional bio-statistical analysis of clinical trial endpoints, with most elements of B set to 0 in the overarching model above. For a single outcome in a mixed effects longitudinal model we will use:

(3) $${\text{Y}}_{\text{ij}}=\left[{\text{β}}_{0}+{\text{β}}_{1}{\text{T}}_{\text{j}}+{\text{β}}_{2}{\text{X}}_{\text{ij}}\right]+\left[{\text{ζ}}_{0\text{i}}+{\text{ζ}}_{1\text{i}}{\text{T}}_{\text{ij}}+{\text{ε}}_{\text{ij}}\right]$$

where Y_ij_ is the outcome measure (Caregiver Burden and Anxiety) for participant (i) at time (j) where j is an indicator for time (baseline, 4 weeks, 8 weeks, 12 weeks, 16 weeks and 20 weeks). β_0_ is the population mean level of the outcome measure prior to the TLC intervention, β_1_ fixed effect for time independent of intervention, X_ij_ is the intervention status (Pre-TLC or Post-TLC/Follow-up) at timepoint j, and β_2_ is the intervention treatment effect. Random effects include individual-level intercept (ζ_0i_), individual-level slope of change over time (ζ_1i_T_ij_), and ε_ij_ for residual error of each outcome measure.

We will then investigate why the overall therapeutic benefit happens, requiring a causal statistical model. The final mediation analysis (H4) suggests the TLC intervention has no direct effect on wellbeing, but acts indirectly by affecting satisfaction with respite time-use. The overall impact comparing wellbeing across the treatment and control groups [E(WB_TLC_)-E(WB_WLC_) from H1] would be explained by this intermediate change. Planned mediational analyses will estimate the magnitudes of each direct and indirect component of the model, with appropriate standard errors [79, 80]. There may be many underlying pathways through which exposure to the full intervention or control/partial condition exerts its effects on wellbeing, but theory as well as our pilot data, identifies this structure as likely to account for the intervention’s anticipated benefits. The empirical test of our explanatory hypotheses is provided by comparing two models, the null hypothesis model lacking a direct effect of the intervention on Wellbeing, and the alternative model allowing such a path: H0: β (intervention **→** wellbeing) = 0. Either outcome is informative.

Although not stated as distinct hypotheses, we will conduct additional exploratory analyses to further explain for whom and how the intervention achieves its effect. These analyses are guided by the following questions: Do variations in the receipt of the intervention modify these relationships? For example, do participants that report higher intervention feasibility (i.e., higher satisfaction and usability of the tool) or higher dosage (i.e, they accessed more of the intervention’s features) show stronger treatment effects? This will be assessed by replacing the dichotomous intervention variable X_ij_ (Group A or Group B) in equation (2) with measures indicating dosage or perceived feasibility. Similarly, we will explore whether individual characteristics or circumstances modify the relationship between intervention, time-use satisfaction, and wellbeing over time. Here, we will use biologic variables of gender and age as well as measures that capture changes in the context of the caregiving relationship as both control variables and interaction terms in the full model expressed in equation (2). Finally, we will explore whether treatment effects maintain, attenuate, or no-change once the intervention period ends. This can be assessed using a repeated-measures design using Group A’s data from immediately post-intervention through the 8-week follow-up period.

These hypothesis driven analyses will focus on the primary outcome measures - Anxiety and Caregiver Burden, but could be repeated with secondary outcomes such as “desire to institutionalize.” Additional analyses could also explore whether and how the intervention may affect outcomes associated with the care-recipient, such as the presence or changes in their behavioral symptoms over time. The TLC study is focused on understanding whether and how the TLC intervention may provide support and benefit to caregivers.

### 3.9 Rigor, Reproducibility, Dissemination

Redeveloping the TLC intervention as a self-administered, technology-facilitated intervention (i.e., an app) increased the accuracy and consistency of how the intervention is delivered. When using trained facilitators to deliver an intervention, explicit strategies are needed to minimize variations and to monitor differences in treatment implementation [56]. Once the TLC app was developed and launched, we did NOT change any of the wording, prompts, or functionality of the TLC intervention, ensuring that every participant received the exact same intervention. Thus, threats to treatment fidelity are minimized, perhaps eliminated, with the standardized and automated messaging provided by an automated app [57], compared to the idiosyncratic nuances associated with human-delivered interventions.

Furthermore, to achieve robust and unbiased results, the TLC study adopted a scientifically rigorous methodology for all phases of the study. For example, even though this is a pilot-test of the TLC intervention, we chose to conduct a full-powered pilot sample that uses randomized assignment and is guided by clear theoretically-informed hypotheses in order to test treatment efficacy, as well as the identification of the mechanisms driving the intervention effect. Using smaller samples and/or less systematic research designs for these early-stage development activities may miss important insight regarding the efficacy and feasibility of the intervention, limiting its ability to be implemented later into practice.

Similarly, we used the five dimensions of the widely-accepted and NIH-endorsed RE-AIM framework (Reach, Efficacy, Adoption, Implementation, Maintenance) [81–85] to guide the overall development and evaluation of the TLC intervention. Prior to the launch of this study, the TLC investigators created, agreed upon, and maintained the RE-AIM checklist and systematically assessed whether the intervention and study design achieved each metric. RE-AIM provides a systematic and standardized way to think about feasibility, and to improve the sustainable adoption and implementation of TLC as an evidence-based intervention. Table 2 shows how we applied and interpreted RE-AIM for purposes of this study.

Throughout the five-year study period (2018–2023), our research team used the Research Electronic Data Capture (REDCap) program to manage screening, enrollment, and retention data, as well as to generate weekly reports to monitor study progress. The REDCap database, created specifically for this project, collected and managed all electronically-administered surveys, allowing us to fully archive all study data and protocols [86] following the completion of the study.

Data archiving and data sharing provide future researchers access to these data: The TLC study is committed to an open-science framework. For example, this report describing the TLC intervention, study-design, and research protocols is being made available via open-access, as a resource to other researchers who may be developing clinical trials methodology for the pilot-testing and evaluation of behavioral interventions for caregivers. All methodology and eventually our results will be recorded within the National Library of Medicine’s www.ClinicalTrials.gov website (NCT03689179). And, lastly, the full TLC data set and all study materials will be archived and made available to other researchers for purposes of replication or education using the University of Utah’s HIVE archive, https://hive.utah.edu.

Finally, in trying to follow best practice for community-engaged research, the TLC research team is committed to giving back to the participants and community partners that assisted in the development of the TLC app and the recruitment of the TLC sample, by providing lay-language presentation of the findings. Throughout the study period, our research team prepared and participated in community-oriented informational sessions, educating communities about dementia and family caregiving, for example. The hyperlink here provides access to a video we created to describe the TLC intervention and preliminary findings to a lay audience; this was sent to all research participants and community partners involved in the study: TLC Summary Video.mp4.

## 4. Discussion

In sum, we developed a comprehensive set of early-stage research activities to redevelop, implement, and pilot-test the TLC intervention, a psychosocial intervention to support ADRD caregivers in maximizing their use of respite time. A focus on respite time-use is not currently present among the many interventions to support family caregivers. Finding ways to support ADRD caregivers - especially with interventions that are scalable, cost-efficient, and developed with the needs of the community in mind - is a national priority and critical to our nation’s public and economic health, given the demographic realities of an aging population and the challenging, often prolonged, caregiving circumstances to those with ADRD.

Unlike many existing caregiver interventions that rely on trained facilitators and controlled settings to establish fidelity, the TLC intervention is a self-administered, technology-facilitated “app” that is cost-efficient to deliver. Committed to community-engaged research practices, we included stakeholder input from the earliest stages of intervention (re)development and throughout the pilot testing of the TLC app, in order to improve translation and scalability to real world practice, with the hopes of accelerating the timeline and success of implementation. We expect that the TLC online intervention, developed in this systematic, yet pragmatic way, will 1) have high practical utility for diverse family caregivers, 2) be a resource that formal respite providers will want to provide to their clients as a cost-efficient and effective source of support that focuses on the caregiver’s respite use and well-being, and 3) elucidate how time-use satisfaction is a mechanism through which respite achieves the intended purpose of maintaining caregiver wellbeing.

## 5. Study Status

Enrollment in the TLC caregiver pilot trial was completed in late fall 2022. Analyses of the TLC pilot-study sample, for purposes of answering the stated aims and hypotheses, will be completed and published during 2024, with additional planned work related to the commercialization and dissemination of TLC for general use to follow.

## Figures and Tables

**Table 1:** Quantifiable Feasibility Indicators and Benchmarks.

Research Feasibility	Benchmark
Length of time to identify potential participants (by recruitment sources)	12 months
Consent/Enrollment Rate vs. those eligible	≥50%
Enrollment rate (participants/month)	≥12/month
Research design-related dropout rate	≤10%
Data collection completion rates (Primary data points and EMAs)	≥80%
**Feasibility**	**Benchmark**
Access to computer & internet	≥90%
Length of time to finalize web page design for testing	24 months
Length of time to hire and train hotline/chat line staff	3 months
System fail rate (e.g., system crashes, down server)	≤5%
Dosage (# of log-ins)	At least 1/week
Completion of intervention protocol (assessment, goal-setting, review)	≥90%
Intervention-related drop-out rate	≤10%
**Acceptability**	**Benchmark**
Caregiver positive or negative appraisal of intervention	≥90% positive
Caregiver confidence that tool will help make better use of respite time (1 = low; 5 = high)	Mean ≥4
Consider using tool in the future (yes/no)	≥90% yes
Would recommend the tool others (yes/no)	≥90% yes
**Usability**	**Benchmark**
On a scale of 1 to 5, How clear were the instructions on the introductory video?	Mean ≥ 4
How easy was it to complete the time-use assessment?	Mean ≥ 4
How easy was it to go through the goal-setting steps?	Mean ≥ 4
How easy was it to complete an action plan to meet your goal(s)?	Mean ≥ 4
How easy was the virtual coach to use?	Mean ≥ 4
How easy was the layout on the screen to follow?	Mean ≥ 4
To what extent was the information presented clear and concise?	Mean ≥ 4
To what extent did having a chatline make the tool more useful??	Mean ≥ 4
To what extent was the chat line easy to use?	Mean ≥ 4
How helpful was the information from the chat line staff?	Mean ≥ 4
How useful were the links to other resources?	Mean ≥ 4
**Implementation/Feasibility (from provider perspective)**	**Benchmark**
On a scale of 1 to 5 Would you consider using this application as part of your service delivery?	Mean ≥ 4
Do you believe your caregiving clients would be interested in using this tool?	Mean ≥ 4
Rank each feature of the tool on its usefulness for your clients.	Mean ≥ 4
In what ways would you incorporate it into how you deliver respite services? What would make you more likely to incorporate this application as part of your service package? What barriers do you believe exist that might make you less likely to incorporate this as part of your service package?	Qualitative examples
What do you think would make your clients more likely to use this tool?	Qualitative examples

**Table 2:** RE-AIM Dimensions, Suggested Questions for Evaluating Intervention, and Proposed Study.

Dimension	Suggested Questions	Proposed Study
Reach	What percent of potentially eligible participants a) were excluded, b) took part and c) how representative were they?	Tracking and analysis of recruitment and eligibility data
Efficacy or Effectiveness	What impact did the intervention have on a) all participants who began the program; b) on process intermediate, and primary outcomes; and c) on both positive and negative (unintended), outcomes including quality of life?	Hypothesis-driven statistical analyses identifying direct and indirect effects of the intervention’s efficacy (Aim 2)
Adoption	What percent of settings and intervention agents within these settings a) were excluded, b) participated and c) how representative were they?	Comparison of provider sample characteristics (Aim 3) to population of respite providers
Implementation	To what extent were the various intervention components delivered as intended, especially when conducted by different (nonresearch) staff members in applied settings?	Qualitative themes describing provider comments about barriers and likelihood of implementation (Aim 3)
Maintenance	What were the long-term effects (minimum of 6–12 months following intervention)?	Initial maintenance or attenuation of intervention efficacy (after 2 months) will be assessed by exploratory analysis comparing post-intervention wellbeing to follow-up wellbeing (Aim 2)
	What was the attrition rate; were drop-outs representative; and how did attrition impact conclusions about effectiveness?	Attrition rates will be calculated and described; missing data will be explored as a potential moderator of intervention efficacy (Aim 2)
	To what extent were different intervention components continued or institutionalized? How was the original program modified?	Stakeholders (Aim 1), caregivers (Aim 2), and providers (Aim 3) will all provide information on how the intervention might be further modified.

Table adapted from: www.re-aim.org.

## Box 1 Study Purpose.

Time for Living and Caring (TLC) is an intervention created to help caregivers maximize the benefits of respite by focusing on their time-use goals. The purpose of this report is to describe the key conceptual features of the TLC intervention, as well as the methodology and protocols used for the (re)development and initial feasibility testing of an online version of the TLC intervention for ADRD caregivers.

## Box 2 Examples of Caregiver Time-Use Goals.

An elderly male caregiver wanted to use respite time to improve his relationship with an estranged adult child. He set a time-use goal to have lunch with said child during an upcoming respite period. This would only be feasible if the adult child was willing and available to meet her during the caregivers’ available respite hours. If this is not feasible, the caregiver might instead write a letter to the child (*compensation*), which could still help increase the likelihood that he would accomplish his underlying goal of improving the strained relationship with his daughter (*optimization*).

A 75-year old female caregiver wanted to use her respite time to re-engage in a neglected hobby – gardening. She had to consider whether she had the financial resources, available time, and physical capabilities to plant a garden in her yard. She identified both physical limitations and financial constraints, which may make the creation of a full backyard garden impractical and infeasible for her. She instead decided to propagate a small windowsill herb garden using a container that she already had. This is an example of *compensation* (still able to garden, but on a smaller level) that facilitates *optimization* (using existing resources and abilities to accomplish the underlying goal of gardening.
